# Unraveling the mitochondrial phylogenetic landscape of Thailand reveals complex admixture and demographic dynamics

**DOI:** 10.1038/s41598-023-47762-w

**Published:** 2023-11-21

**Authors:** Kitipong Jaisamut, Rachtipan Pitiwararom, Poonyapat Sukawutthiya, Tikumphorn Sathirapatya, Hasnee Noh, Wikanda Worrapitirungsi, Kornkiat Vongpaisarnsin

**Affiliations:** 1 Forensic Genetics Research Unit, Department of Forensic Medicine, Faculty of Medicine, Chulalongkorn University, Bangkok, Thailand; 2 Department of Forensic Medicine, Faculty of Medicine, Chulalongkorn University, Bangkok, Thailand; 3 Forensic Serology and DNA, King Chulalongkorn Memorial Hospital and Thai Red Cross Society, Bangkok, Thailand

**Keywords:** Anthropology, Population genetics

## Abstract

The evolutionary dynamics of mitochondrial DNA within the Thai population were comprehensively explored with a specific focus on the influence of South Asian admixture. A total of 166 samples were collected through randomized sampling, ensuring a diverse representation. Our findings unveil substantial genetic and haplogroup diversity within the Thai population. We have identified 164 haplotypes categorized into 97 haplogroups, with a notable inclusion of 20 novel haplogroups. The distribution of haplogroups exhibited variations across different populations and countries. The central Thai population displayed a high diversity of haplogroups from both the M and N clades. Maternal lineage affinities were discerned between several Mainland Southeast Asia (MSEA) and South Asian populations, implying ancestral genetic connections and a substantial influence of South Asian women in establishing these relationships. *f*_*4*_-statistics indicates the presence of a Tibeto-Burman genetic component within the Mon population from Thailand. New findings demonstrate two phases of population expansion occurring 22,000–26,000 and 2500–3800 years ago, coinciding with the Last Glacial Maximum, and Neolithic demographic transition, respectively. This research significantly enhances our understanding of the maternal genetic history of Thailand and MSEA, emphasizing the influence of South Asian admixture. Moreover, it underscores the critical role of prior information, such as mutation rates, within the Bayesian framework for accurate estimation of coalescence times and inferring demographic history.

## Introduction

Mainland Southeast Asia (MSEA) has a rich and complex history. Archaeological evidence indicates that anatomically modern humans inhabited the region for at least 50,000 years^1,2^, and recent research suggests that habitation may date back as far as 86,000 years^3^. Numerous archaeological sites, dating back to 10,000–45,000 years ago, have been well documented throughout the area^4^. During the late Pleistocene to Holocene periods, the Hoabinhian cultural system was widespread among hunter-gatherer groups spanning from southern China to Sumatra^5^. However, only two ancient DNA samples have been recovered from Pha Faen, Laos (7888 ± 40 years before the present (YBP)) and Gua Cha, Malaysia (4319 ± 64 YBP)^6^ and modeled as a deeply diverged East Eurasian lineage. Furthermore, the genetic data revealed a relationship with modern Andamanese hunter-gatherers such as Önge and MSEA Negritos, including Jehai^6,7^. The Neolithic population exhibits an admixed genetic profile between a deeply diverged east Eurasian lineage of hunter-gatherers and East Asian of southern Chinese agriculturalists, which is like modern-day Austroasiatic-speaking populations such as Mlabri^6,7^. The Bronze Age in MSEA emerged in the late second millennium BCE, with the earliest evidence of bronze metallurgy dating to around 1100 BCE, reflecting continued cultural interaction with southern China^8^. The genetic study of Bronze Age individuals from Northern Vietnam found substantial genetic turnover suggesting an additional wave of migration from Southern China during the Bronze and Iron Ages^6,7^. In the late first millennium BCE, the region was incorporated into the Maritime Silk Road through connections between regional networks linking Southeast Asia, China, South Asia, and beyond. The presence of walled urban settlements which are associated with centralized policies and can be classified as incipient states such as Angkor Borei, Oc Eo, and Khao Sam Kaeo, dating back to the fourth century BCE, indicates the existence of well-established and dynamic local and regional networks that facilitated integration^9^. The cultural interaction between South Asian and local MSEA populations created synergies that influenced early state formation in MSEA^9^.

Due to prehistoric and continuous human habitation, the region exhibits significant ethnolinguistic and genetic diversity. Specifically, Thailand, located in the center of MSEA, is home to approximately 68.61 million people who speak 73 recognized languages from five linguistic families: Kra-Dai, Austroasiatic, Sino-Tibetan, Austronesian, and Hmong-Mien^10^. Kutanan et al.^11^ initially analyzed 1234 complete mtDNA sequences from 51 Kra-Dai and Austroasiatic groups in Thailand and Laos, revealing high genetic heterogeneity across the region with 212 haplogroups. Significant genetic differentiation existed among samples from the same ethnolinguistic group. Notably, Kra-Dai groups exhibited more genetic homogeneity than Austroasiatic groups, which displayed a higher proportion of basal mtDNA lineages. In a subsequent study, Kutanan et al.^12^ analyzed an additional 560 complete mtDNA sequences from 22 distinct populations, leading to the identification of 62 new haplogroups. Researchers also found West Eurasian-associated haplogroups in some populations in MSEA, which could be a signal of South Asian admixture^12^. Previous genome-wide studies had documented the presence of South Asian genetic admixture in MSEA populations particularly those with strong cultural influences from India including the Bamar, Cham, Khmer, Malay, Mon, and Thai^13–16^. However, it is absent in present-day hunter-gatherer groups or relatively isolated groups from the highlands of northern Thailand^13^.

**Figure 1:**
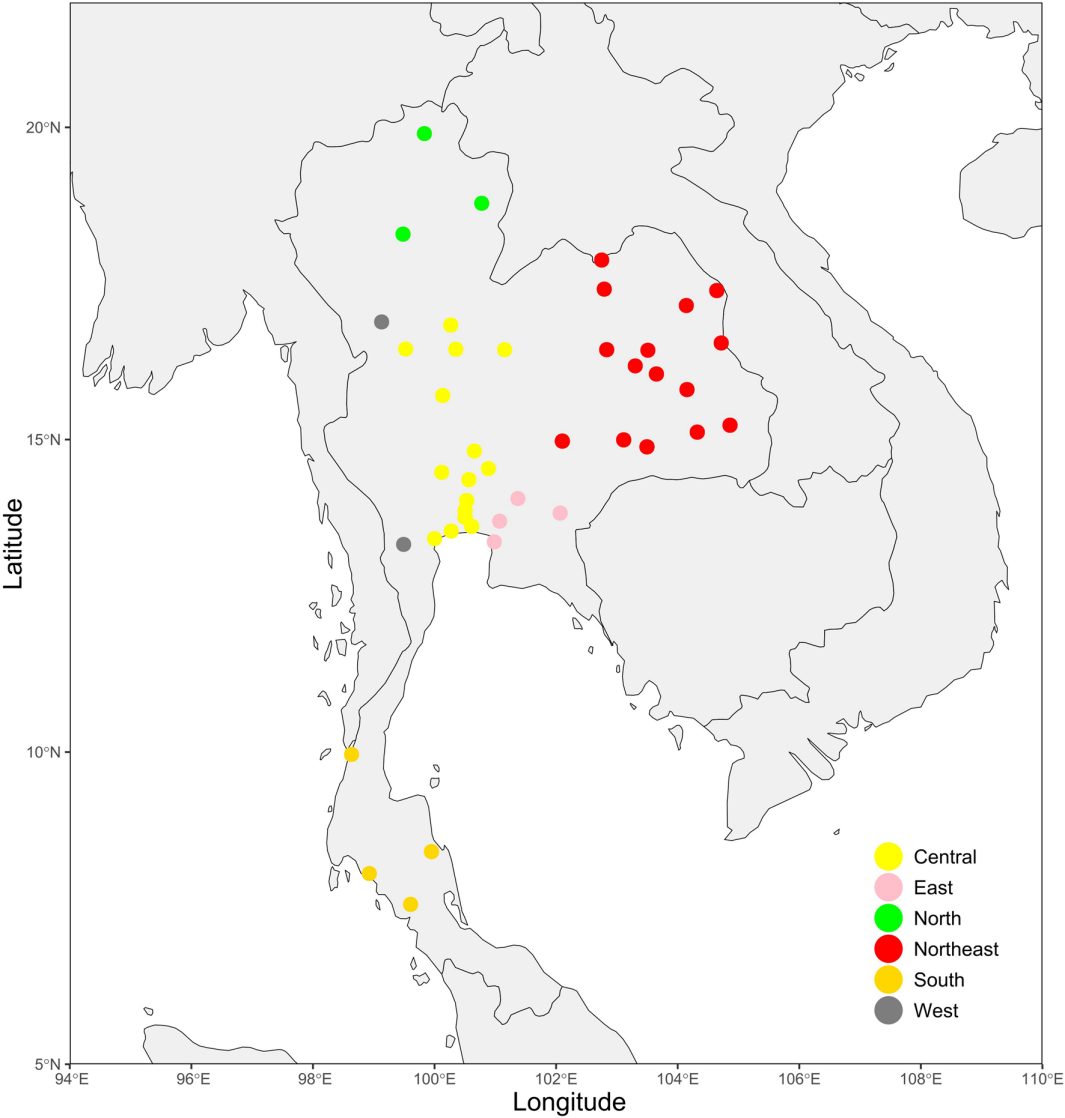
Geographic locations of populations for whom mitochondrial data were generated in this study. The map was plotted using an R package “*rnaturalearth*” (https://github.com/ropensci/rnaturalearth) with Natural Earth map data (https://www.naturalearthdata.com/).

Recent archaeological discoveries and investigations into South Asian admixture in MSEA populations have highlighted the present-day Thai population’s complex mtDNA evolutionary history and diversity. This study aims to expand our knowledge of Thailand’s mtDNA landscape by comprehensively analyzing an additional 166 whole mtDNA sequences from the randomized samples of self-identified Thais, regardless of their ethnic backgrounds. This approach will minimize potential biases associated with targeting specific groups, providing a more comprehensive overview of the genetic diversity of the present-day Thai population. By examining the data in conjunction with previous studies and considering South Asian admixture, this study will achieve a more comprehensive understanding of the complex evolutionary history of mtDNA in Thailand (Fig. 1).

## Results

### Haplogroup assignment and statistical analysis

**Figure 2:**
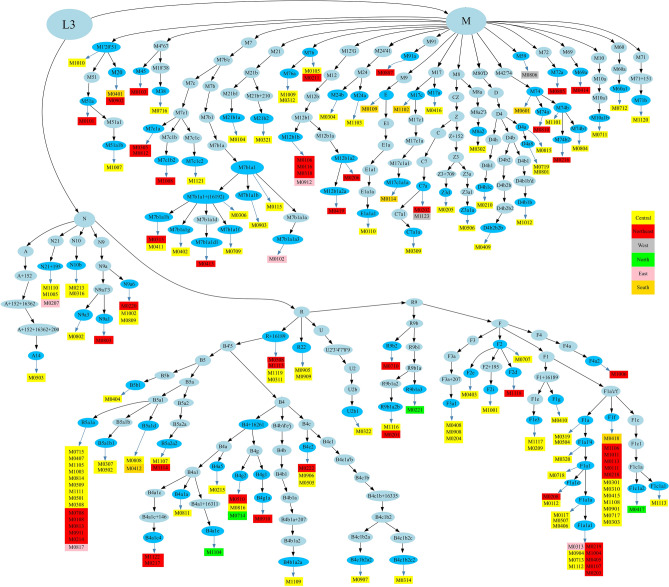
Phylogenetic representation of 166 mtDNA samples. Haplogroups can be terminal (blue, oval shape) or intermediate nodes (light blue, oval shape), while rectangles indicate the samples under the corresponding haplogroup, with color-coding according to the geographic regions of the samples, as indicated in the legend on the right.

A total of 166 randomly selected samples including 143 males and 23 females whose ages ranged from 3 to 87 years (an average of 46 years, s.d. = 16.94) were grouped by Thai government administrative region. Samples are from Bangkok and Central (n = 99, 59.64%), Northeast (n = 49, 29.52%), South (n = 7, 4.22%), East (n = 5, 3.01%), North (n = 4, 2.41%), and West (n = 2, 1.20%) (Supplementary Table S1). We generated 166 complete mtDNA sequences with mean coverages ranging from 54 × to 3687 × . The overall genetic and haplogroup diversities of the Thai population were 0.999 ± 0.0007 and 0.98 ± 0.0050, respectively (Table 1). Specifically, high levels of genetic diversity were observed in all regions studied, indicating a remarkably diverse Thai population. In a previous study by Kutanan et al.^11^, the use of ethnolinguistics classification resulted in a wider range of haplogroup diversity, which ranged from 0.8 to 1.0. We observed 164 haplotypes among the 166 sequences that could be classified into 97 haplogroups, of which 20 haplogroups (Supplementary Table S1) were not observed in previous studies^11,12,17^. The samples-to-haplogroup ratio which was calculated by dividing the number of samples by the number of subhaplogroups was 1.71 (166 samples/97 haplogroups) while the previous studies were 5.82 (1234 samples/212 haplogroups)^11^, and 3.22 (560 samples/174 haplogroups)^12^ respectively. A higher ratio implies that a larger number of samples is used to identify a smaller number of haplogroups, indicating lower genetic diversity or greater relatedness among the samples. The haplogroups observed belong to macrohaplogroup M (n = 63, 37.95%), R9 (n = 48, 28.92%), B (n = 48, 28.92%), and N (n = 11, 6.63%), respectively. The most frequent haplogroups are B5a1a (n = 15, 9.04%), F1f. (n = 13, 7.83%), and F1a1a1 (n = 9, 5.42%). We employed *HaploGrep2* to generate DOT graphs represented in Fig. 2 which clearly illustrates the phylogenetic tree of all samples. We noticed that macrohaplogroup R had more significant clusters (with B5a1, F1a, and F1f. being the prominent clades) compared to macrohaplogroup M, where M7b1a1 was the largest clade with fewer samples. A similar pattern was observed in Thai and Cambodian populations in previous studies^11,18^. The phylogenetic tree generated by Bayesian Evolutionary Analysis Sampling Trees (BEAST) reveals the distinctions among individual samples in B5a1, F1a, and F1f. clades (Supplementary Figs. S1, S2). This observation highlights the limited phylogenetic resolution of these three groups, as suggested by Kloss-Brandstätter et al.^18^. B4′5 consists of two sister clades, B4 and B5, which have high frequencies in MSEA (24% in the present study). It was noted that all samples collected from the northern region of Thailand were assigned to the R clade, while most of the samples from the southern region fell under the M clade (Fig. 2). Previous research has indicated that distinguishing between the northern and southern Thai subpopulations is possible by employing a set of 273 ancestry informative markers (AIMs)^19^. However, it is important to acknowledge that the sample sizes from both regions in this study were limited, preventing definitive conclusions regarding possible variations in maternal lineage demographics. The haplogroups from the M clade, such as M7, M8, and M9, as well as haplogroups from the N clade, including R9, R11, and R22, have been identified among Central Thai individuals (Fig. 2). This diversity suggests that multiple maternal lineages have participated in the population's history, likely through historical migrations, interactions, and genetic exchanges within and beyond the region.

**Table 1:** Population information and summary statistics (number of polymorphic sites (S), haplotype diversity (*h*), mean number of pairwise differences (MPD), nucleotide diversity (π), standard deviation (s.d.)).

Population	n	Number of haplotypes	S	*h* (s.d.)	MPD (s.d.)	π (s.d.)	Number of haplogroups	Haplogroups diversity (s.d.)
Central	99	99	689	1 (0.0007)	38.736 (16.975)	2.34E−03 (1.14E−03)	68	0.98 (0.006)
East	5	5	96	1 (0.0014)	29.167 (16.293)	1.76E−03 (1.18E−03)	5	1 (0.130)
North	4	4	56	1 (0.1265)	37.630 (16.645)	2.27E−03 (1.12E−03)	4	1 (0.180)
Northeast	49	49	393	1 (0.1768)	36.762 (18.275)	2.22E−03 (1.26E−03)	32	0.97 (0.012)
South	7	7	114	1 (0.0041)	33.000 (23.685)	1.99E−03 (2.02E−03)	7	1 (0.080)
West	2	2	33	1 (0.5000)	38.250 (16.710)	2.31E−03 (1.12E−03)	2	1 (0.500)
Overall	166	164	911	0.999 (0.0007)	38.736 (16.975)	2.34E−03 (1.14E−03)	97	0.98 (0.005)

Calibrating the tips of the BEAST phylogenetic tree with ancient samples allows for more reliable and informative evolutionary analyses. This approach was adopted in this study by incorporating 20 additional ancient samples (Supplementary Table S2). The coalescence times for individual haplogroups differ from previous studies^11,12^ when using a single mutation of 4.33 × 10^−8^ site^−1^ year^−1^ to the entire mtDNA sequence without partitioning. Notably, our study reveals higher coalescence times compared to previous research. However, coalescent ages of haplogroup became closer and comparable with previous studies when we applied the same partition scheme and mutation rates to analysis (partitioning the mtDNA into coding and non-coding regions with mutation rates of 1.708 × 10^−8^ and 9.883 × 10^−8^, respectively) (see Supplementary Table S3.)

Analysis of Molecular Variance (AMOVA) revealed that only 0.4% of the total genetic variation was attributable to differences between populations classified by geographic regions. However, the observed genetic differentiation (Phi value = 0.13) was not statistically significant at the 0.05 significance level (Supplementary Table S4). This suggests that the observed genetic differentiation between populations is likely due to random chance or stochastic processes. This finding is consistent with previous studies that applied language family and geographic classification, which reported proportions of variation between groups of 0.91% (*P* < 0.01) and 0.17% (*P* > 0.01), respectively^12^. These results suggest that the language family may be a better indicator of the genetic structure of Thai/Lao populations than geography. However, we caution that language family and geographic classification may be poor indicators of genetic structure due to the high levels of variation within populations and the low levels of variation between populations. Furthermore, a Mantel test for correlations between genetic and geographic distances indicated no correlation (r = − 0.04315, *P* > 0.01) supporting the limited impact of geography on the genetic structure of populations in Thailand.

### Relationship to global populations

**Figure 3:**
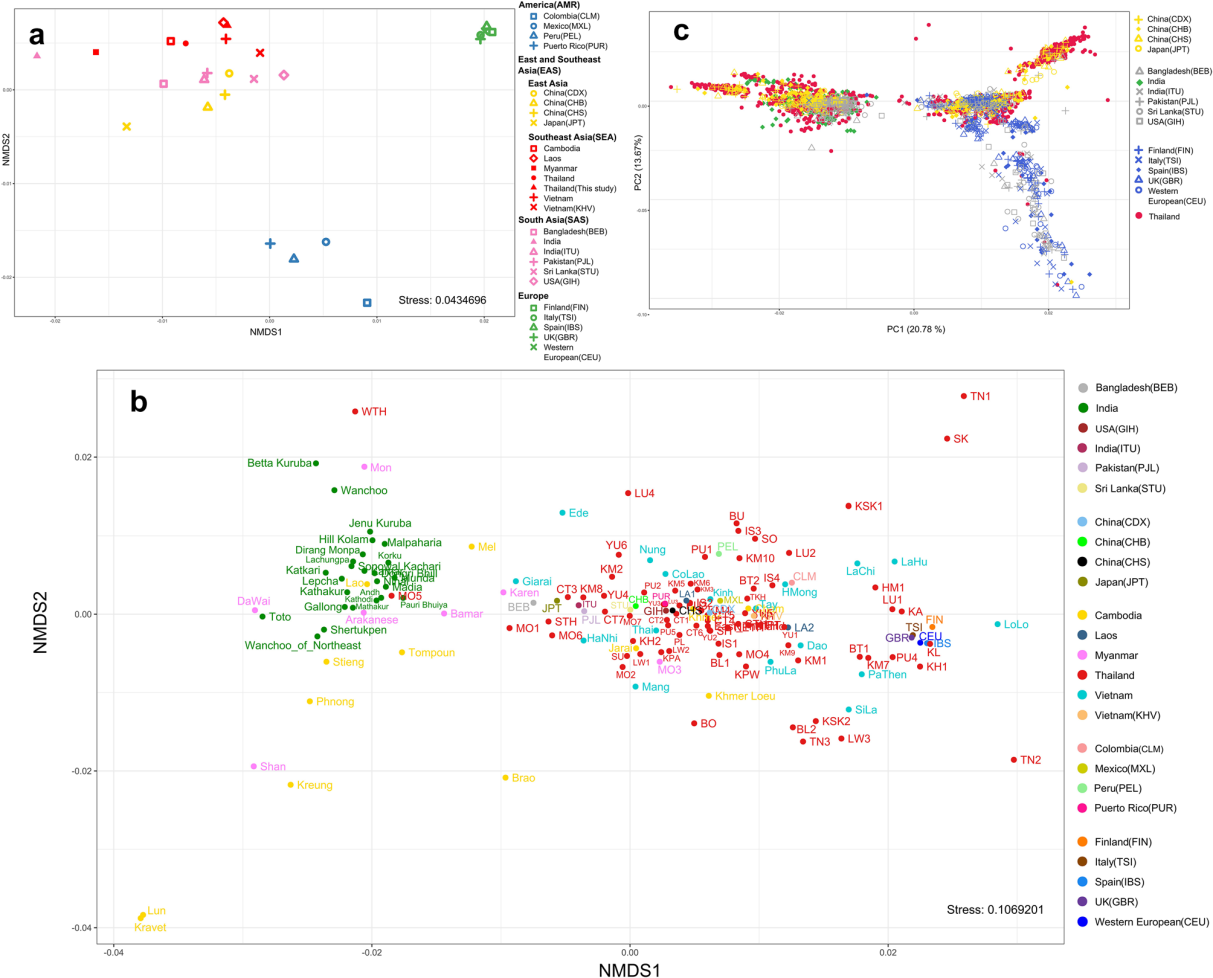
This figure provides visual representations of the genetic relationships among populations at different levels. (**a**) NMDS plot at the global population level (**b**) The NMDS plot highlights the genetic relationships at the population level (**c**) Principal component analysis (PCA) plot at the individual level, specifically highlighting the variation captured by PC1 and PC2. These plots are based on a genetic distance matrix derived from mtDNA genomes. All details of populations and abbreviations can be found in Supplementary Table S5.

To examine the relationship of our data to other populations, we initially generated a nonmetric multidimensional scaling (NMDS) plot at the global population level (Fig. 3a). African populations appeared isolated and distinct from other populations, so we excluded them from subsequent analyses (Supplementary Fig. S3). At the global population level, populations from macrogeographic regions such as South Asia, East Asia, Southeast Asia, the Americas, and Europe exhibited a high level of affinity and clustered together. China and Japan displayed closer relationships with South Asia than with Southeast Asia. India emerged as a distinct and unique entity, standing apart from other South Asians. Subsequent analysis at the population level (Fig. 3b) revealed a diminished discriminatory power. As expected, a considerable number of populations from Myanmar, namely Bamar, Arakanese, Da Wai, and Mon, exhibited pronounced affinities with Indian populations(green), as evident from their coherent clustering (Fig. 3b). Tibetan-related ancestry in the Burmese population was inferred using *qpGraph* and *SOURCEFIND* analysis and South Asia admixture was dated back to 443–466 YBP^13^. Cambodian populations, including Stieng, Lao, Tompoun, Phnong, and Mel, exhibited notable affinities with Indian populations. Most Thai groups exhibited distinct clustering apart from India, except for MON5, which notably clustered with Indian populations. Additionally, the WTH displayed a partial inclination toward the Indian cluster, though to a lesser extent compared to MON5. Among the populations from Vietnam, Giarai was the solitary group showing an inclination toward the Indian cluster. To investigate the individual-level relationships between populations, a principal component analysis (PCA) was performed, and the result is shown in Fig. 3c. Thai and East Asian populations share a similar pattern, as they span the PC1 axis, suggesting a higher level of genetic diversity compared to Europe, which forms a distinct cluster along the PC2 axis. Interestingly, India (ITU) clusters together with Europe, while India (green) demonstrates a separate genetic position. For Thailand, three main clusters are discernible. The first cluster aligns with East Asia and India, indicating potential genetic affinities and shared ancestry. The second cluster includes other South Asian countries, East Asia, and Europe. The final cluster comprises exclusively populations from East Asia, suggesting a distinct genetic makeup.

### Relationship with populations in South Asia and East Asia

**Figure 4:**
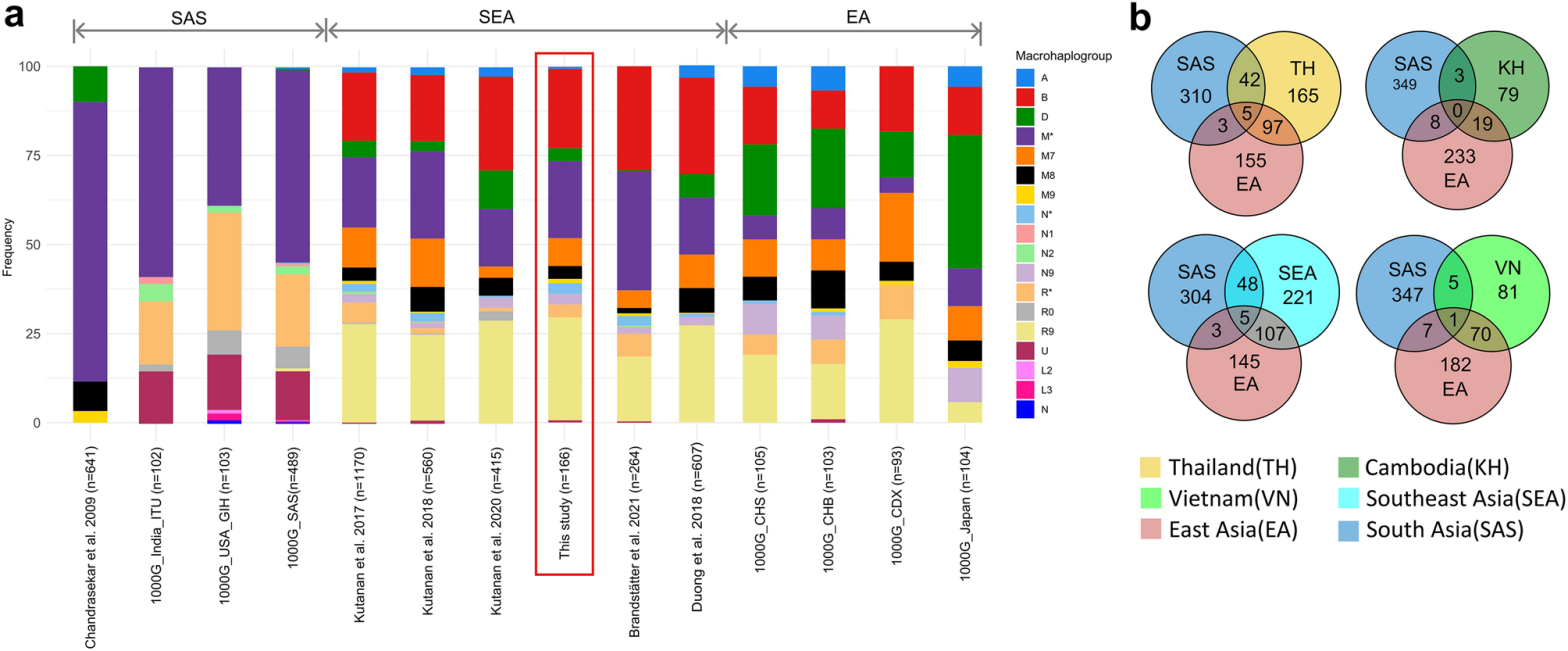
(**a**) Haplogroup distribution in various data sets including the 1000 Genome Project data. The distribution of haplogroups is categorized according to publications, with a primary focus on SEA populations, including the current study's data set, Kutanan et al.^11,12,17^ (mostly Thailand), and Kloss-Brandstätter et al.^18^ (Cambodia); South Asia (SAS), including Chandrasekar et al.^21^; and East Asia (EA), including Chinese Dai in Xishuangbanna (CDX), Han Chinese in Beijing (CHB), and Southern Han Chinese (CHS). (**b**) A Venn diagram elucidates the extent of haplogroup sharing based on the terminal lineage among diverse populations.

The maternal genetic relationship between Thai populations and other populations in South Asia and East Asia is complex and multifaceted. Figure 4a,b provide complementary perspectives on the genetic diversity of these populations. Figure 4a provides a comprehensive overview of the macrohaplogroup distribution across different datasets, while Fig. 4b focuses on the shared haplogroups among different populations, defined based on the terminal lineage. Figure 4a shows that haplogroup M is dominant in Indian populations and accounts for a significant portion of Thai and Cambodian samples as well. East Asians, in contrast, exhibit lower frequencies of M7, M8, and M9 haplogroups compared to other South Asian and Southeast Asian populations. Haplogroup A is prevalent in East Asian populations, but the Chinese Dai population in Xishuangbanna (CDX) shows a unique distribution pattern that has a greater similarity with Southeast Asian populations. This observation highlights the magnitude of regional genetic variations within East Asians and emphasizes the influence of population dynamics in terms of historical migrations. Haplogroup D is not exclusive to any region. It is prevalent in North Asia (11–34%), Central Asia (14–20%), East Asia (10–43%), and Southeast Asia (5–10%)^20^. Thailand has been found to show a notable prevalence of haplogroup D in all studies (Fig. 4a). However, a contrasting pattern is observed in Cambodia, where the presence of haplogroup D is comparatively limited. This discrepancy in the distribution of haplogroup D between Thailand and Cambodia implies divergent maternal lineage demographic patterns associated with East Asian ancestry in these respective populations. This difference also suggests that Thailand and Cambodia could experience different patterns of migration and admixture from East Asia.

**Figure 5:**
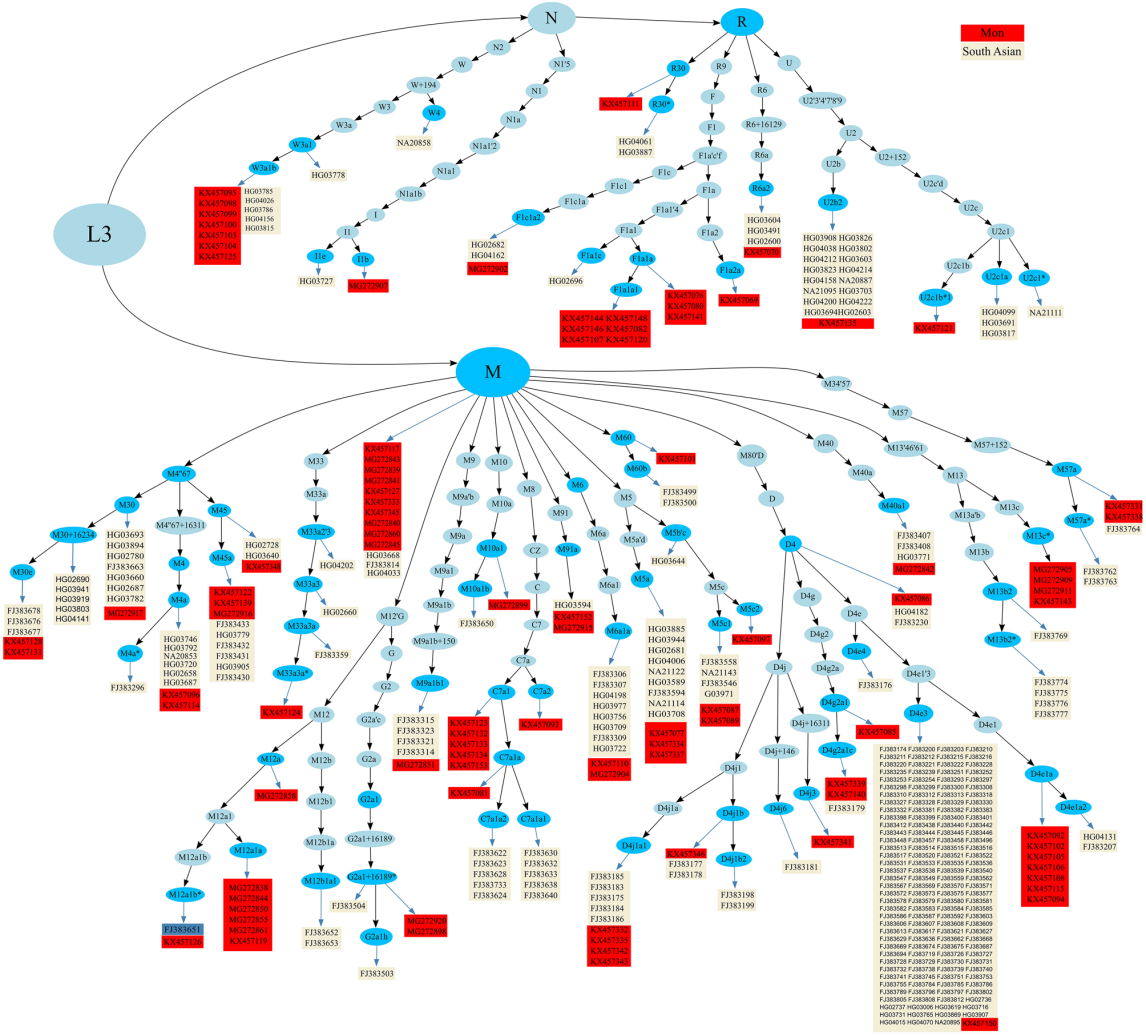
Shows a phylogenetic representation of Mon and South Asian populations. Haplogroups are classified into terminal (blue oval shape) or intermediate (light blue oval shape) nodes. Rectangles represent samples falling within the respective haplogroup, color-coded to indicate populations (red for Mon and dark blue for South Asian).

Although South Asia populations possess a smaller number of macro-haplogroups, they exhibit a remarkable diversity of mtDNA haplogroups, predominantly falling within the clade M (Fig. 4a,b). This higher abundance of haplogroups in South Asians highlights the maternal lineages’ distinctiveness. Southeast Asian populations display a mixture of maternal lineages from South Asians and East Asians, with South Asians primarily contributing to the prevalence of macro-haplogroups within clade M, while East Asians exhibit greater prominence in macro-haplogroups within clade R. Haplogroup sharing analysis reveals that Thailand share approximately 33% haplogroups with East Asia and 15% with South Asia, signifying a notable genetic affinity with both regions (Fig. 4b). Similarly, Vietnam and Cambodia exhibit a stronger genetic connection to East Asian than South Asian. Thai populations display the highest degree of haplogroups sharing with South Asians compared to Cambodian and Vietnamese populations (Fig. 4b). Among Thai populations, the Mon group exhibits the highest proportion of shared haplogroups with South Asians, accounting for approximately 34% (Supplementary Table S6). This finding aligns with previous research emphasizing their genetic affinity^11,13^. To investigate this relationship more comprehensively, we compiled a dataset consisting of 1288 samples from both the Mon and South Asian populations and generated a phylogenetic tree using *Haplogrep2*. To avoid burdening readers with the complexities of the complete phylogenetic tree, we chose to focus on a pertinent section that highlights the key findings (Fig. 5). Mon exhibits shared basal haplogroups and subhaplogroups with the South Asian populations and highlights the presence of significant genetic diversity. D4j1a1, M45a, and W3a1b are notable among the shared haplogroups, with the D4 lineage demonstrating the highest prevalence. Within the D4 lineage, 18 individuals from the Mon population are distributed across various subhaplogroups, including D4j1a1, D4e1, and D4e1a2. These findings emphasize the existence of distinct genetic substructures within the primary haplogroups and suggest a certain degree of genetic connection and shared ancestry between these populations.

### Demographic history of the populations in Thailand

**Figure 6:**
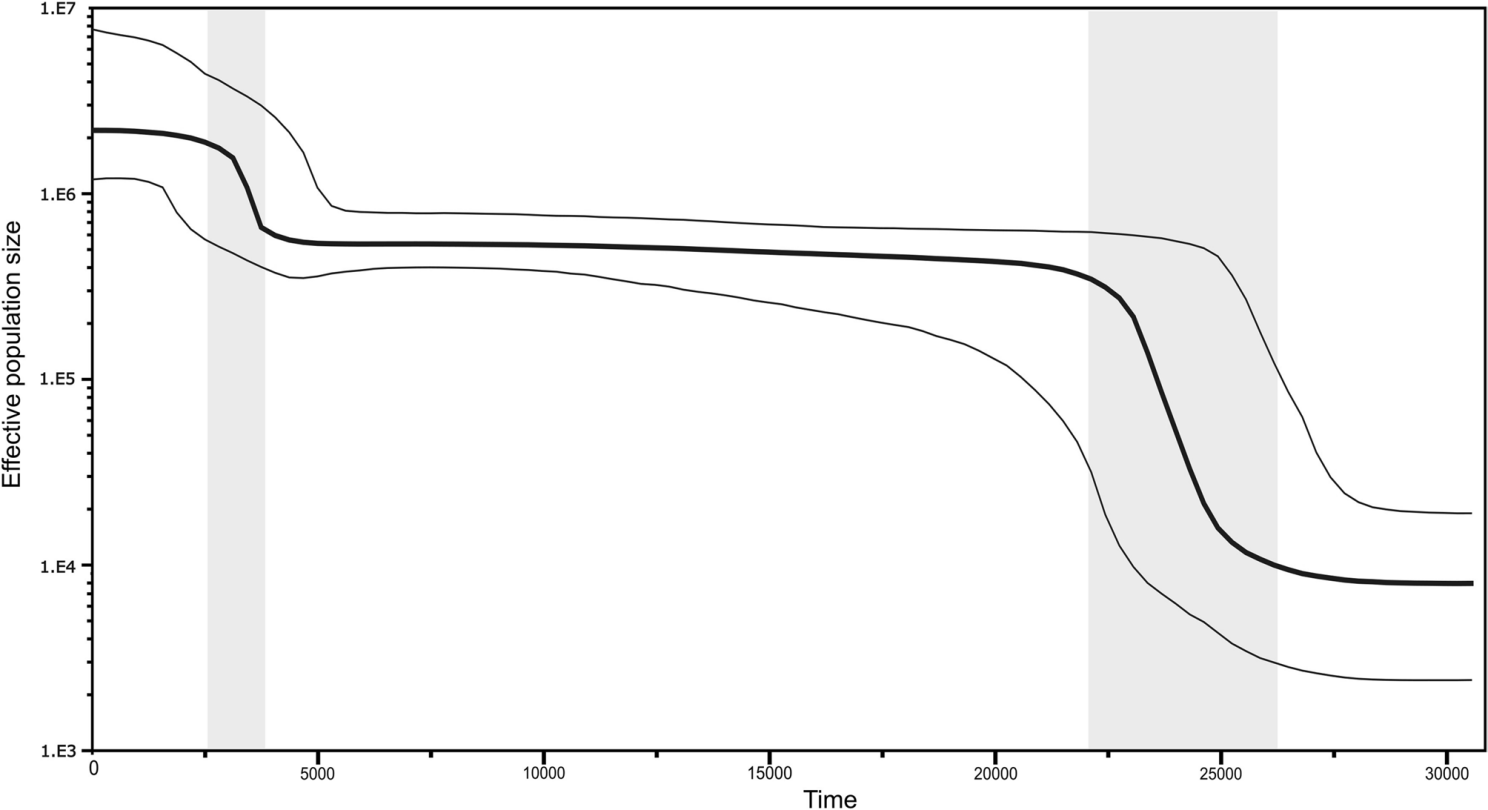
The Bayesian skyline plot illustrates the estimated *N*_*e*_ over time in years before the present when applying a mutation rate of 4.33 × 10^−8^ substitutions site^−1^ year^−1^ without mtDNA partitioning. The thick black line represents the median estimate, while the two thin lines correspond to the upper and lower bounds of the 95% highest posterior density (HPD) interval. The x-axis denotes the time in YBP, and the y-axis is presented on a logarithmic scale.

The Bayesian skyline plot (BSP) is a valuable tool for visualizing the demographic history of populations based on changes in effective population size (*N*_*e*_) over time. In Fig. 6, the plot reveals valuable information about the demographic history of Thailand. It demonstrates the initial period of population expansion occurring between approximately 26,000 and 22,000 YBP when *N*_*e*_ underwent a substantial and remarkable increase. In particular, the *N*_*e*_ value exhibited a more than 20-fold surge, highlighting a significant population expansion event. After the initial expansion, *N*_*e*_ remained stable until approximately 3800 YBP, after which another growth phase was observed. This subsequent growth phase persisted from approximately 3800 YBP to 2500 YBP, during which *N*_*e*_ experienced a notable fourfold increase. Interestingly, from 2000 YBP to the present, the *N*_*e*_ exhibited a stable trend. These findings suggest a complex demographic history characterized by different population dynamics. The initial population expansion, followed by a period of stability, and the subsequent phase of growth indicate intricate patterns of population growth and contraction. The stable population size observed in recent history suggests a relative equilibrium in the population dynamics.

## Discussion

The primary objective of this study was to enhance our understanding of the evolutionary patterns of mtDNA in the Thai population and its correlations with neighboring countries, while also taking into consideration the impact of South Asian admixture. We observed a significant cluster that includes the haplogroups B5a1, F1a, f1f., and M7b1a1 (Fig. 2). Notably, the B5a1 and F1a haplogroups comprise a substantial proportion of the Cambodian samples, representing 38% in a previous study^18^ and 21% in our study. This suggests the presence of an autochthonous population in Cambodia. The high prevalence of B5a1 and F1a haplogroups in both populations indicates a potential origin of these haplogroups in Cambodia, followed by subsequent expansions into other regions, including Thailand. This could be attributed to historical migrations, interactions, or genetic admixture events between the Cambodian and Thai populations, leading to an increased frequency of individuals carrying the B5a1 and F1a haplogroups. Multiple Cambodian populations forming clusters alongside Indian populations (Fig. 3b) suggest a significant maternal genetic affinity. Nevertheless, the shared haplogroups between them are limited to three (Fig. 4b). The disparity between NMDS (Fig. 3b) and haplogroup sharing (Fig. 4b) highlights complex population interactions. NMDS clustering can be shaped by diverse factors, including ancient ancestry, genetic drift, migration, and recent admixture. In contrast, haplogroup sharing reflects more recent genetic admixture. The lack of haplogroup sharing implies that while there might be common ancestry or genetic influence, specific genetic lineages have not been shared in recent times. Changmai et al.^13^ elucidated the South Asian admixture in the Khmer population (indigenous people of Cambodia) approximately 7.2% using *f*-statistic-based *Admixtool2* with autosomal SNPs and estimated the admixture date around 4 CE during the Angkor period using haplotype-based *Globetrotter*. However, a subsequent study found significant South Asian admixture (ca. 40–50%) in a protohistoric individual from the Vat Komnou cemetery at the Angkor Borei site in Cambodia. Radiocarbon dating places this individual in the early period of Funan (1 CE), suggesting the possibility of South Asian migration to MSEA and intermarriage with local populations before or during the early stage of state formation^14^. This study provides additional evidence from mtDNA that contemporary Cambodian populations carry genetic contributions from South Asian ancestry. Moreover, the result suggests a substantial contribution of South Asian women to the genetic makeup of present-day Cambodian populations.

The Ede and Giarai are two distinct populations residing in Vietnam, all of which have Austronesian language roots due to cultural diffusion^22^. Machold et al.^23^ found that the Y-haplogroups R1a-M420 and R2-M479, associated with west Eurasia, occurred at low frequencies in the Ede population (8.3% and 4.2%, respectively) and in the Giarai population (3.7% and 3.7%). Changmai et al.^13^ using *qpAdm*, *qpGraph*, and *SOURCEFIND* methods, consistently inferred the presence of South Asian ancestry in the Ede and Giarai populations (7.5 ± 2.1%, 7.4 ± 2.0% respectively, as inferred by *qpAdm)*. The comprehensive genetic evidence derived from Y-haplogroups, and autosomal DNA admixture strongly supports the relationship between the Ede, Giarai, and South Asian populations^13,23^. The NMDS plot (Fig. 3b) positions Ede and Giarai relatively closer to South Asian populations compared to other Vietnamese groups. However, the gap between them remains wider than seen in Cambodian and Myanmar populations. Therefore, our findings suggest that while Ede and Giarai show some maternal lineage affinity with South Asians, mtDNA evidence alone is insufficient to decisively establish a conclusion about the contribution of South Asian women to their genetic makeup.

Building upon the data obtained from the study conducted by Kutanan et al.^11^, we have taken an additional step by visualizing and representing the genetic association between Mon and South Asian populations in a phylogenetic tree. Interestingly, Mon and Punjabi individuals in Lahore, Pakistan, share several haplogroups in the M clade, including M, M30, M45, M4a, and M5a, suggesting a common ancestral population. Mon also shares some haplogroups in the M clade, such as M40a1, M45a, and M5c1, with the Indian Telugu population in the UK. The Telugu-speaking people primarily reside in the states of Andhra Pradesh and Telangana in South India, while Punjabi-speaking people are predominantly found in the Punjab region spanning across India and Pakistan. It is surprising to note that Mon harbors haplogroups from both the South and North Indian subcontinents. The *qpAdm* analysis infers approximately 12% South Asian ancestry within the Mon population^13^. Additionally, the Mon group exhibits minor occurrences of West Eurasia-associated haplogroups, including J (5%) and R (16%)^24^. Furthermore, we observed that Mon shares specific haplogroups within the D clade (including D4j1a1, D4j1b, and D4g2a1c), with the Wanchoo people (an indigenous Tibeto-Burman ethnic group residing in the Northeast Indian state of Arunachal Pradesh), as well as with the Gallong people (a small tribal group in the West Siang district of Arunachal Pradesh, who speak a Tibeto-Burman language)^25^. This observation aligns with the findings of Changmai et al.^13^ and Kutanan et al.^15^, who reported the presence of additional ancestry from a Tibetan-related source in the Mon population. They hypothesized that this Tibetan-related ancestry in Mon might have been acquired through interactions with Sino-Tibetan speaking populations in Myanmar. Based on the aforementioned observations, we have performed additional analysis to further investigate the relationship between the Mon population and Sino-Tibetan speaking populations applying *f*_*4*_-statistics in the form of *f*_*4*_(Tibeto-Burman, Dai; Mons, other Thailand groups) and *f*_*4*_(Tibeto-Burman, Han; Mons, other Thailand groups). The result of *f*_*4*_-statistics (Supplementary Figs. S4–S5) supports the existence of the Tibeto-Burman genetic component in Mon populations as all *f*_*4*_-statistic values are positive (|Z|> 3 in most cases), which indicates that the Tibeto-Burman groups share more genetic drift with Mons than with other Thailand groups. In this study, we also found that Sino-Tibetan shared 12.5% mtDNA haplogroups (4 shared haplogroups/32 total haplogroups) with Mon. This proportion is similar to the autosomal SNPs that Mon and Sino-Tibetan showed an admixture proportion of around 10% when inferred using SOURCEFIND^13^.

Our study identified two phases of population expansion in Thailand: an initial phase of around 22,000–26,000 YBP and a second phase of around 3800 YBP. The initial phase contrasts with a previous study that proposed an expansion of around 55,000 YBP while the second phase has not been reported in the previous study^11^. The discrepancy in timing can be attributed to the variation in mitochondrial mutation rates and mtDNA partition schemes applied in the respective studies. The mutation rate of mtDNA is not constant but fluctuates over time, influenced by factors such as population size and dynamics. In growing populations, there is a higher number of lineages, leading to an increased accumulation of mutations per lineage. These lineages also persist for longer periods, resulting in an accelerated evolutionary rate that surpasses the mutation rate. Conversely, in declining populations, the evolutionary rate decreases.

We used a single mutation rate of 4.33 × 10^−8^ substitutions per site per year based on a recent study^26^. However, there is no consensus on the best partitioning scheme and mutation rates to use in coalescent dating and BSP analyses. To assess the sensitivity of our results to the choice of partitioning scheme and mutation rate, we performed two additional analyses. The first analysis used a single model with a single mutation rate of 2.285 × 10^−8^ site^−1^ year^−1^, which was previously applied by Maier et al.^27^. This analysis showed that the timeframes shifted to around 48,000 YBP for the initial expansion and 7500 YBP for the second expansion (Supplementary Fig. S6a). The second analysis partitioned mtDNA into coding and non-coding regions and applied mutation rates of 1.708 × 10^−8^ and 9.883 × 10^−8^ respectively. This analysis shifted the timeframe for the initial expansion to around 53,000 YBP, close to the estimation of Kutanan et al.^11,12^ (Supplementary Fig. S6b). Bayesian methods provide a probabilistic framework for estimating coalescence times and inferring demographic history through the integration of prior information, such as mutation rates and evolutionary models, with observed genetic data. The mutation rate shapes outcomes noticeably, as evidenced by our empirical findings presented in Fig. 6, Supplementary Fig. S6a,b, and Supplementary Table S3. Diverse studies have adopted different mtDNA partitions and mutation rates, yielding varied results. When estimating the age of the haplogroup, a higher mutation rate leads to older estimates, while a lower rate produces younger ones. Similarly, in the BSP, the mutation rate affects calibrating the molecular clock used to estimate demographic features like population size changes over time. The critical role of mutation rates and partition schemes in these analyses highlights the necessity for accurate estimation. However, precise estimation is challenging due to its fluctuating nature, resulting in various rates adopted across studies. This variability can lead to variations in age estimates and demographic insights. This predicament prompts us to explore innovative solutions.

The initial population expansion phase (Fig. 6) corresponds to the Last Glacial Maximum (LGM, 19,000–26,500 years ago)^28^. During this period, Earth underwent intense cold and widespread glaciation, affecting numerous regions and leading to harsh environmental conditions. However, Southeast Asia remained relatively more habitable. Furthermore, the sea level dropped significantly, reaching a minimum level of ca. 120 m below the present level^29^. The lowered sea level led to the emergence of habitable lands with abundant resources, creating favorable conditions for the expansion and admixture of modern human populations^30^. This environmental change facilitated migration across Southeast Asia^31^. Following the LGM (around 18,000 years ago), the flooding of the Sunda shelf (marine transgression)^32,33^ likely caused a contraction of inhabitable areas towards inland regions, stabilizing population expansion^34,35^. During LGM, several new haplogroups emerged, providing additional evidence for population expansion. Notable among them are the Southeast Asian lineage M91 (25,600 years ago), the Northeast Indian haplogroup M49e (25,100 years ago), M49e1 (20,200 years ago), and Indochina R9b (19,000 years ago)^36,37^. We also observed a second phase of population expansion occurring between 3800 and 2500 YBP, which had not been previously documented in studies on mtDNA in the Thai population. Moreover, the sudden increases in effective population size might indicate the arrival of new groups or gene flow from neighboring regions. Gignoux et al.^38^ demonstrated a global Neolithic expansion through mitochondrial lineage analysis. They also indicated that the population size of Southeast Asia began to expand around 4700 years ago (CI 3000–5700 years ago), which aligns with our findings. This period corresponds to the Neolithic demographic transition, characterized by the adoption of agriculture by prehistoric societies, resulting in increased food production and decreased mobility compared to foraging practices. In Southeast Asia, this transition took place between 2500 and 1500 BCE and brought about significant changes in the biological, linguistic, and cultural evolution of the region^39^. Around 6000–4000 BCE, rice (*Oryza sativa japonica*) was fully domesticated in the mid-lower Yangtze River valley of central China^40^. Subsequently, rice and millet farmers from the Yangtze and Yellow River regions migrated southward through different routes, reaching Baiyangcun in Yunnan around 2650 BCE^41^. By 2200–2000 BCE, they had reached coastal Vietnam and Thailand, and by 1700–1500 BCE, they had expanded to the interior Khorat Plateau, as reported by Higham et al.^42^. Archaeological excavations have yielded artifacts such as domestic rice, pottery styles, and tools, demonstrating a connection to southern China. Notable examples include Khok Phanom Di (ca. 2000–1500 BCE), Ban Chiang, and Non Nok (ca. 1100–1000 BCE) as well as Tha Kae and Khok Charoen (ca. 2000/1800–1100 BCE)^43^. In addition to archaeological findings, the studies on autosomal DNA have provided supporting evidence for a subsequent increase in the effective population size. Neolithic populations in MSEA were a mixture of deeply diverged East Eurasian lineages and East Asian agriculturalists who migrated from South China approximately 4000 years ago^6,7^ and the genetic structure of Bronze Age individuals from Northern Vietnam (ca. 2000 years ago) suggests another migration wave from southern China to MSEA^6,7^.

Our study provides insights into the mtDNA) landscape of the present-day Thai population and its connections with neighboring regions. However, some findings are based on the neutral theory of molecular evolution such as BSP, which has been challenged by many authors^44–48^ while the maximum genetic diversity theory is an alternative framework. Therefore, we approach our conclusions with caution.

In summary, our study investigated mtDNA evolution within the Thai population, focusing on South Asian admixture. We identified 20 novel haplogroups from 166 randomly selected individuals. Haplogroup distribution was found to vary across populations and countries. Maternal lineage affinities connect MSEA and South Asian populations, highlighting ancestral genetic association and the significant influence of South Asian women. Notably, *f*_*4*_-statistics indicate a Tibeto-Burman genetic element in Thailand's Mon population. Our findings indicate two population expansion phases, aligning with the LGM and Neolithic transition. Importantly, this work enhances our understanding of the mtDNA landscape of Thailand. Moreover, it also emphasizes the critical role of prior knowledge in the Bayesian framework for accurate inference of demographic history.

## Material and methods

### Sample collection

This study was conducted to investigate the mtDNA landscape of the Thai population in strict adherence to ethical guidelines and principles. All experimental protocols received approval from the Institutional Review Board (IRB) of the Faculty of Medicine, Chulalongkorn University (IRB No. 817/64). The need for informed consent was waived by the IRB of the Faculty of Medicine, Chulalongkorn University under 45CFR46.101(b), as all 166 blood samples were taken from medicolegal autopsies authorized by the Faculty of Medicine, Chulalongkorn University (Certificate of Exemption No. 049/2021). The Thai ethnicity of the deceased individuals was recorded according to their identity cards, which were provided by their relatives.

The sampling frame for this study was all deceased individuals who underwent a medicolegal autopsy at the Faculty of Medicine, Chulalongkorn University in 2019 and 2020. We employed a randomized sampling approach to minimize bias. The samples were randomly selected from the available medicolegal autopsy cases. This approach allowed us to collect samples without any specific bias toward gender, geographic location, or ethnolinguistic background. By doing so, we aimed to minimize the associated bias and the risk of selecting samples that might not accurately represent the overall population. We were able to collect 166 samples from the sampling frame. The sample consisted of 143 males and 23 females, with an age range of 3–87 years (mean: 46 years, standard deviation: 16.94 years). We acknowledged that gender imbalance in our sample could influence the representation of certain maternal lineages in our study.

### DNA extraction and long-range PCR

DNA extraction using the QIAamp DNA Blood Mini Kit (Qiagen). All dsDNA was quantified using NanoDrop™ 2000 spectrophotometers (Thermo Scientific) and diluted to 0.1 ng/µl. A long-length amplification was performed with two sets of specific primer pairs (forward 1:9396-416; reverse 1:1878-92 and forward 2:15195-216; reverse 2:9777-97) using the LA PCR Kit (TaKaRa) following the manufacturer’s protocol. The PCR products were qualified and quantified by Agarose gel electrophoresis and NanoDrop™ 2000 spectrophotometers, respectively. These PCR products for each sample were diluted to 0.2 ng/µl and pooled in 1 well with 10 µl for each PCR product.

### Library preparation and massively parallel sequencing

DNA libraries were prepared using Nextera XT DNA Library Preparation Kit for the first 90 samples and Nextera DNA Flex Library Preparation Kit (Illumina) for another 78 samples with 1 ng of DNA input. Library preparations were performed following the manufacturer’s protocols with a bead-based normalization method using 1% spiked-in PhiX control v3 (Illumina). Pool DNA libraries were sequenced by MiseqFGx (Illumina) using Miseq Reagent Nano Kit v2 (300 cycles) with a 2 × 150 bps read length.

### Data analysis and haplogroup assignment

The sequencing data were aligned with the revised Cambridge Reference Sequence (rCRS; NC_012920.1), mapping and determining variants using mtDNA Variant Processor and mtDNA Variant Analyzer v1.0.0 (Illumina). MtDNA haplotypes were assigned to haplogroups based on PhyloTree Build 17^49,50^ with *HaploGrep2*^51^ following the established criteria of EMPOP.

### Statistical analysis

To investigate the mtDNA landscape of the Thai population, we grouped samples into six administrative regions and plotted their geographic locations to determine data distribution (Fig. 1). We observed variation in sample size across regions but could not balance the samples due to the limitation of medicolegal autopsy cases. We determined the overall population summary statistics and for each region to provide an overview of genetic diversity, especially for the regions with a high number of samples. Various statistical methods were applied to investigate genetic diversity, including measures of haplotype diversity, nucleotide diversity, pairwise genetic distances between populations, and the Mantel test to determine the correlation between genetic and geographic distances between populations. These analyses were performed using standard libraries in R^52^, including *geodist*, *vegan*^53^, *Ape*^54^, *Adegenet*^55^, and *Ade4*^56^. The results were visualized using the *ggplot2*^57^ package. We acknowledge that our results may be influenced by ethnolinguistic background, gender ratio imbalance, and sample size bias. We are aware that some regions have few samples, and our interpretations of findings from these regions should be approached with caution, as they may not accurately represent the regional population. Findings from these regions should be considered preliminary and ideally validated through larger-scale or targeted group research to ensure the reliability and validity of the results.

### Nonmetric multidimensional scaling (NMDS)

To provide a comprehensive population representation, a dataset of 6533 complete mtDNA sequences was compiled, incorporating 166 newly generated data from our study along with 2504 samples from the 1000 Genome Project Phase 3 as provided by Weissensteiner^18^, and data from previous studies^11,12,17,18,21,36,58,59^, which all detail can be found in Supplementary Table S5. NMDS is a technique used to simplify multivariate data into a few important axes, allowing for easier recognition and interpretation of patterns and differences among groups. In this study, NMDS analysis was performed on the combined dataset using a genetic distance value with the function “*dist.genpop*” from the *Adegenet*^55^ package and then applying the “*metaMDS*” function from the *Vegan*^53^ package in R. The results were visualized using the *ggplot2* package. The application of NMDS allowed the identification of patterns and differences among these populations, further contributing to our understanding of the genetic diversity in this region.

### Principal component analysis (PCA)

PCA was performed to observe a cluster of samples following to mitochondrial sequencing variant using *smartpca*^60^ version 18,140 from the *EIGENSOFT* package (https://github.com/DReichLab/EIG). The computed principal components (PC) for populations from East Asia, Europe, South Asia, and Thailand were analyzed and visualized using the *ggplot2* package in R.

### Phylogenetic reconstruction and divergence time dating

The phylogenetic tree was generated directly with *HaploGrep2* based on PhyloTree Build 17. The “*lineage*” function generated a DOT file that was visualized with Graphviz and adapted in Inkscape. We complemented the *HaploGrep2* tree with Bayesian inference using BEAST^61^ to ensure consistent topologies. Multiple sequence alignment of sequences to rCRS using Geneious Prime. All data conversion, alignment, and file formatting steps were performed in Geneious Prime. Software jModel test 2.1.7^62^ was used to select the most suitable model during the creation of the input file for BEAST using BEAUTi v1.8^63^. We applied the HKY + Γ model to our data. Exponential priors were employed to specify the prior distributions for the gamma shape parameters in the analysis. Additionally, lognormal priors were utilized to define prior distributions for the kappa parameters of the HKY model. These choices were made based on the previous study^27^ to incorporate prior information and assumptions regarding the parameter values. The strict clock model was tested in our analysis, utilizing a lognormal prior distribution for the mutation rate (μ). Numerous mutation rates have been published (Supplementary Table S7). However, there is no consensus on the best partitioning scheme and mutation rate to use in coalescent dating and BSP analyses. Some studies have partitioned mtDNA into coding and non-coding regions with respective mutation rates of 1.708 × 10^−8^ and 9.883 × 10^−8^ substitutions per site per year^11,12,58^, while others have partitioned mtDNA into 4 subsets and used different mutation rates for each region^64–66^. Still, others have used one average mutation rate for whole mtDNA sequences^27,67^. We chose to consider the entire mtDNA sequence and use a single mutation rate of 4.33 × 10^−8^ substitutions site^−1^ year^−1^ based on a previous study which reported the time dependency effect on the evolutionary rate with increasing acceleration in recent times and observed mutation rate ranging from 1.91 × 10^–8^ (95% CI 1.72–2.10 × 10^–8^) mutations site^−1^ year^−1^ for the oldest period (40,160 ± 4658 years ago) to 4.33 × 10^–8^ (95% CI 3.90–4.82 × 10^–8^) for the most recent period^26^. We reasoned that this mutation rate is appropriate for our study because we study present-day populations. We also performed two additional analyses to assess the sensitivity of our results to the choice of partitioning scheme and mutation rate. The first analysis used a single model with a single mutation rate of 2.285 × 10^−8^ per site per year, which is an average of mtDNA mutation rates reported in the literature^64,66,68–72^ and applied by Maier et al.^27^. The second analysis partitioned mtDNA into coding and non-coding regions and applied mutation rates of 1.708 × 10^−8^ and 9.883 × 10^−8^ respectively. It is the same approach applied by Kutanan et al.^11,12^ in the studies of populations in Thailand. The results of these additional analyses are presented in Supplementary Table S3 and Supplementary Fig. S6. For the tree priori, we selected a non-parametric Bayesian skyline model so as not to assume anything a priori about population size or tree shape through time.

In BEAST, tip date calibration refers to the process of assigning dates or age estimates to the tips (terminal nodes) of the phylogenetic tree based on known or estimated dates of those samples. BEAST used Bayesian inference to estimate the posterior distribution of parameters, including divergence times, substitution rates, and tree topology, given the input data and prior knowledge Tip date calibration has been reporting no significant influence on the reconstructed topology but has a notable effect on substitution rate and divergence time estimates^66^. Therefore, to achieve the calibration of the phylogenetic tree, 20 ancient samples with associated tip ages measured in calibrated years before the present (calBP) were assigned based on dates available from previous studies^71,73–79^. By incorporating such reliable temporal information, we were able to enhance the accuracy and reliability of the inferred evolutionary relationships within the studied mtDNA lineages. We performed 4 × 10^7^ MCMC steps by sampling every 4 × 10^3^ steps. Tracer^80^ was used to visualize and diagnose MCMC output and to assess a stationary distribution of posterior samples, within and between MCMC chains. We used several tools from the BEAST package to analyze and visualize the posterior tree samples. First, we used *LogCombiner* to combine the posterior tree samples generated from the MCMC analysis. This step allowed us to merge the individual trees into a single file for further analysis. Next, we summarized the combined tree samples using *TreeAnnotator*, another component of the BEAST package. *TreeAnnotator* enabled us to derive the maximum clade credibility (MCC) tree, which represents a summary tree that captures the most credible evolutionary relationships among the sampled sequences. To visualize and interpret the results, we utilized *iTOL*; therefore, we were able to depict and analyze the MCC tree, facilitating the interpretation of the evolutionary relationships and providing a visual representation of the inferred phylogenetic patterns.

In this study, we employed a Bayesian Skyline analysis, which utilizes a Markov Chain Monte Carlo (MCMC) approach, to estimate *N*_*e*_ over time based on the number of coalescent events. It is important to note that the inferred population demography obtained from the BSP analysis primarily reflects the dynamics of the samples included in the analysis. Therefore, to focus specifically on the population dynamics of Thailand, the 20 ancient samples that originated from regions outside of Thailand were excluded from the analysis. To ensure an accurate estimation, the BSP was conducted using a substantial number of MCMC chains, totaling 40,000,000. A burn-in period of 4,000,000 generations was implemented and excluded from the analysis to allow the chains to reach a state of convergence. By reconstructing the population's demographic history through time, we can gain valuable insights into the fluctuations in *N*_*e*_ and better understand the underlying processes that have influenced the population dynamics in Thailand.

### f_4_-statistics

*F*_*4*_-statistics analysis was performed using the Diploid genome-wide SNP. The dataset was derived from the Affymetrix Human Origins SNP "array and merged with previously published ancient and present-day population data. Detailed information regarding the SNP data can be found in the original article^13^. To calculate the *f*_*4*_-statistics, we employed the “*qpdstat*” function from the R package “*ADMIXTOOLS2*”^81^ with default settings (https://uqrmaie1.github.io/admixtools/index.html). We focused on two specific population combinations for our analysis: *f*_*4*_(Tibeto-Burman, Dai; Mons, other Thailand groups) and *f*_*4*_(Tibeto-Burman, Han; Mons, other Thailand groups). To visualize the results, we generated plots in Supplementary Figs. S4–S5 using the *ggplot2* package in R.

### Supplementary Information

Supplementary Information.

Supplementary Tables.

## Data Availability

The 166 mitochondrial genome sequences reported in this study are deposited in NCBI GenBank under accession numbers mentioned in population metadata (Supplementary Table S1).
