# Supplementary material for: Unraveling the mitochondrial phylogenetic landscape of Thailand reveals complex admixture and demographic dynamics
Source: Sci Rep. 2023 Nov 21;13:20396. doi: 10.1038/s41598-023-47762-w (PMC10663463; doi:10.1038/s41598-023-47762-w)
Supplement: Supplementary file 1 — Supplementary Information. [file 41598_2023_47762_MOESM1_ESM.docx]

# Supplemental Material

**Unraveling the Mitochondrial Phylogenetic Landscape of Thailand Reveals Complex Admixture and Demographic Dynamics**

The supplemental material consists of Supplementary Figures S1-S6 and legends for supplementary Tables S1-S7. The Tables are provided in a separate Excel file.

**Supplementary Figures: pages 2-7**

**Supplementary Tables Legends: pages 8-9**

**Supplementary Tables: see separated Excel file**


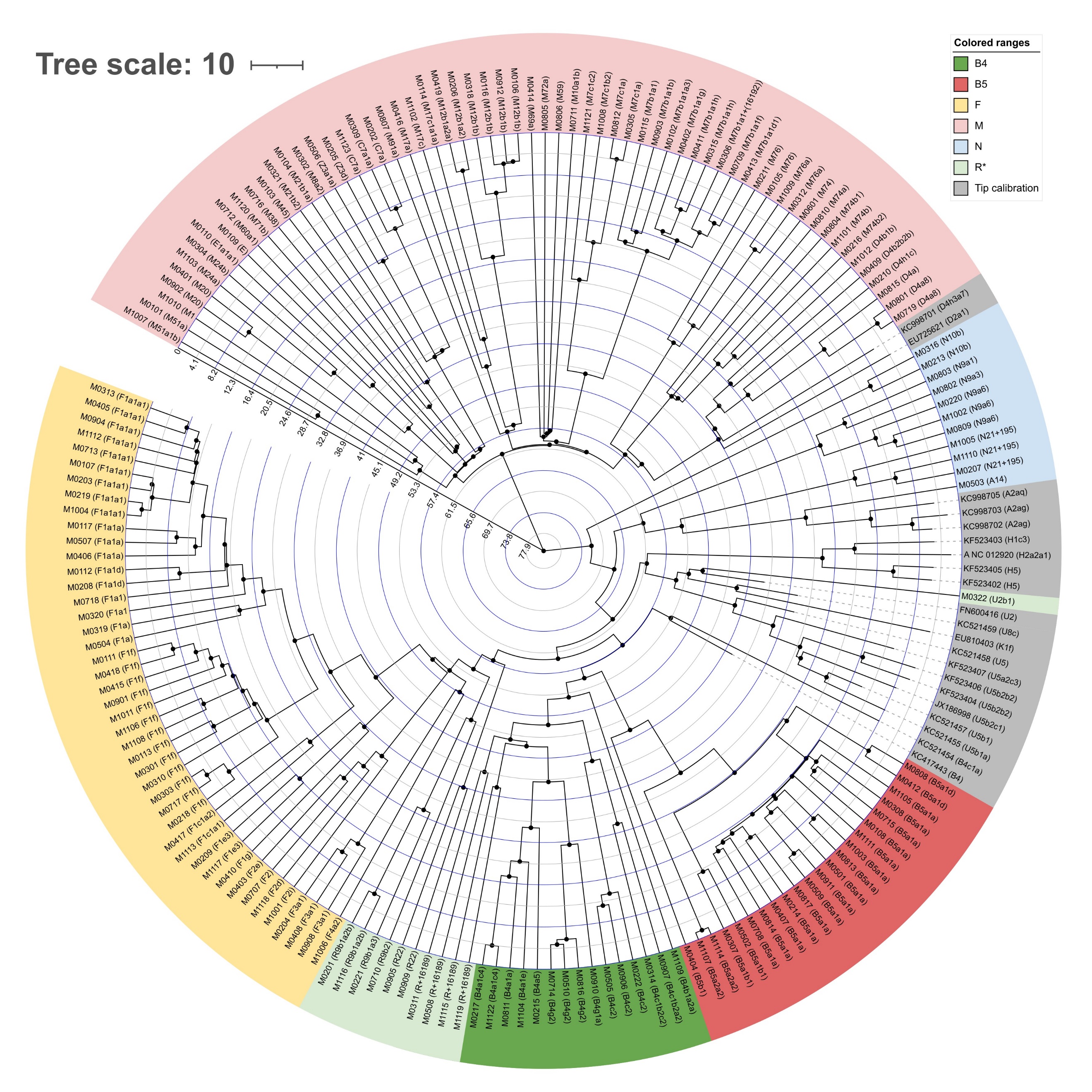


**Supplementary Figure S1**: Bayesian maximum clade credibility (MCC) tree of 187 mtDNA samples, including those from this study and tip calibration samples, generated by BEAST based on a multiple sequence alignment of the sequences to the reference sequence (rCRS) using Geneious Prime. The non-partition mtDNA sequence and single mutation rate of 4.33 × 10^−8^ substitutions site^-1^ year^-1^ were applied. The figure was generated in iTOL.


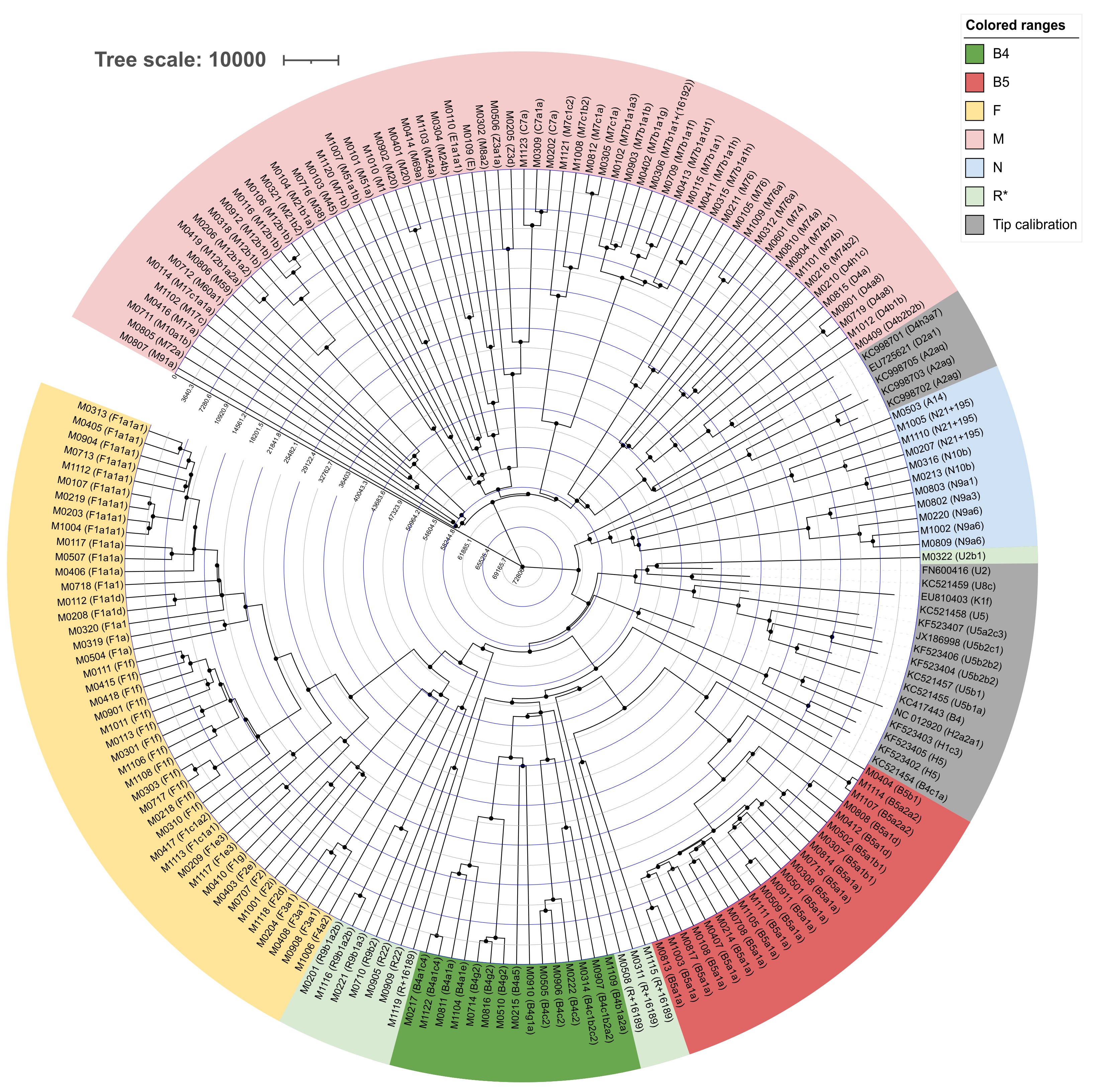


**Supplementary Figure S2**: Bayesian maximum clade credibility (MCC) tree of 187 mtDNA samples, including those from this study and tip calibration samples, generated by BEAST based on a multiple sequence alignment of the sequences to the reference sequence (rCRS) using Geneious Prime. The partition of mtDNA sequence into coding and non-coding regions with mutation rates of 1.708 × 10^−8^ and 9.883 × 10^−8^ respectively substitutions site^-1^ year^-1^ were applied. The figure was generated in iTOL.


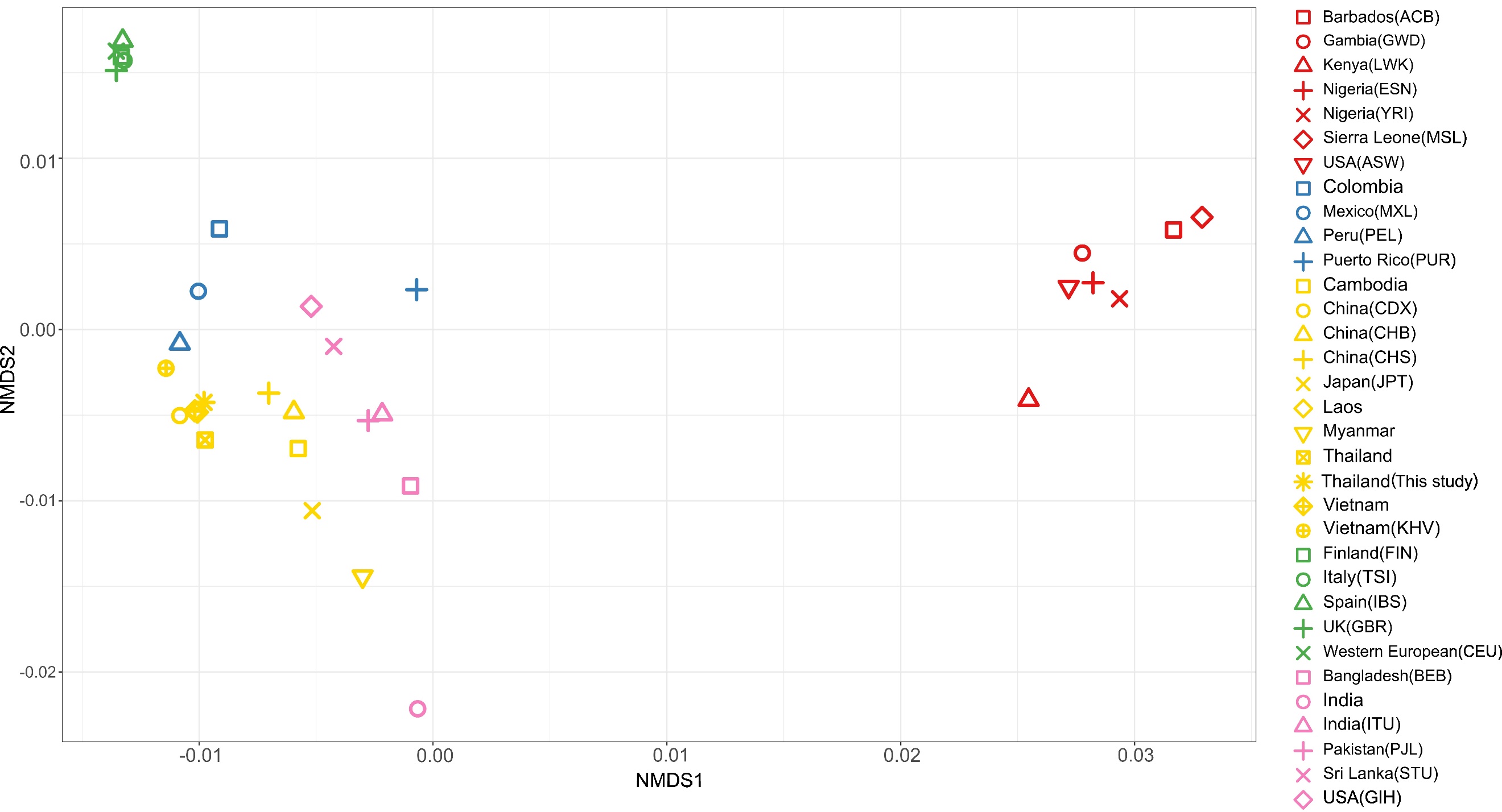


**Supplementary Figure S3.** Non-metric multidimensional scaling (NMDS) plot of the genetic relationships among global populations, including African populations.


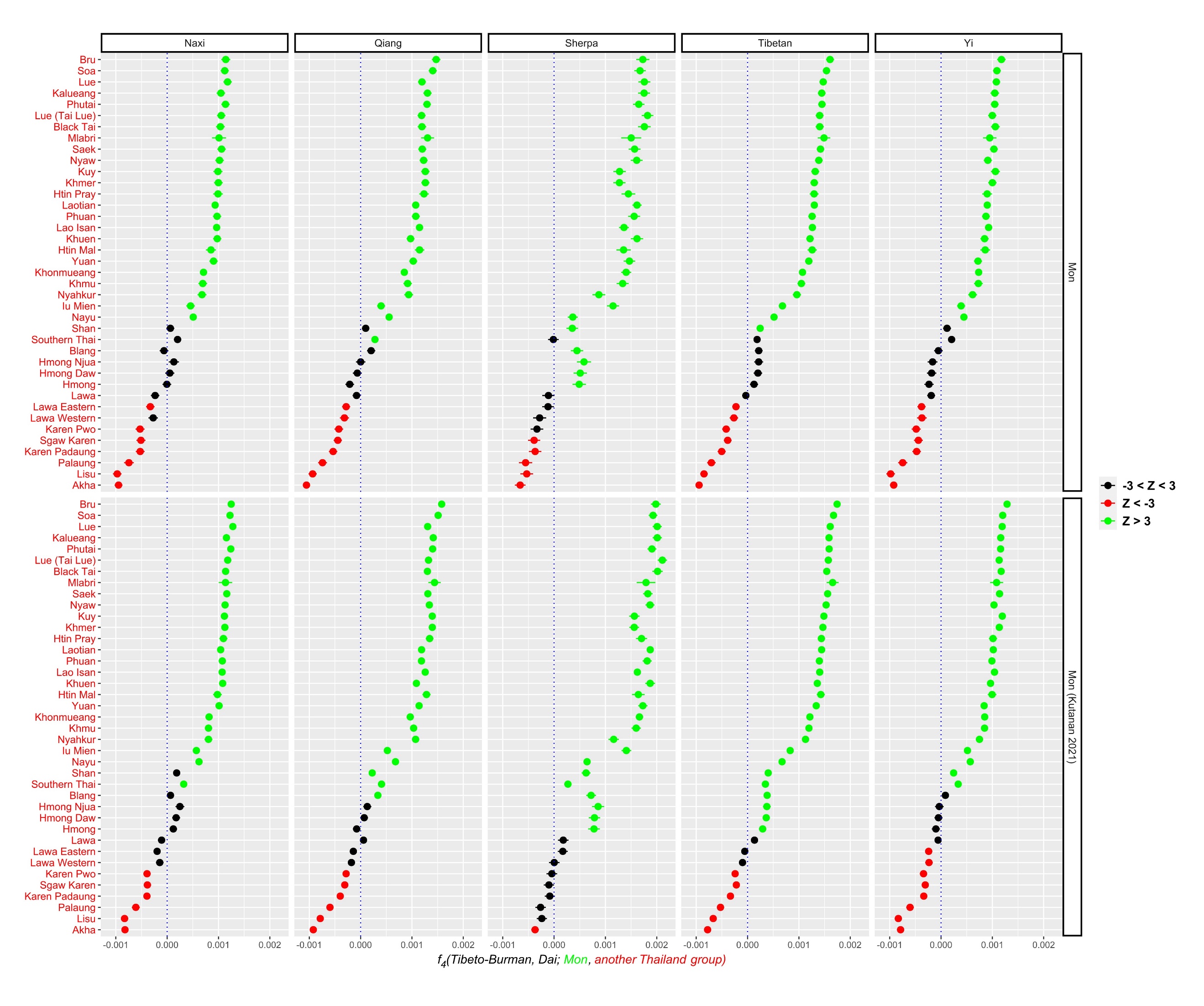


**Supplementary Figure S4.** Plot of *f*_4_ statistics value of *f*_4_(Tibeto-Burman, Dai; Mons, other Thailand groups)


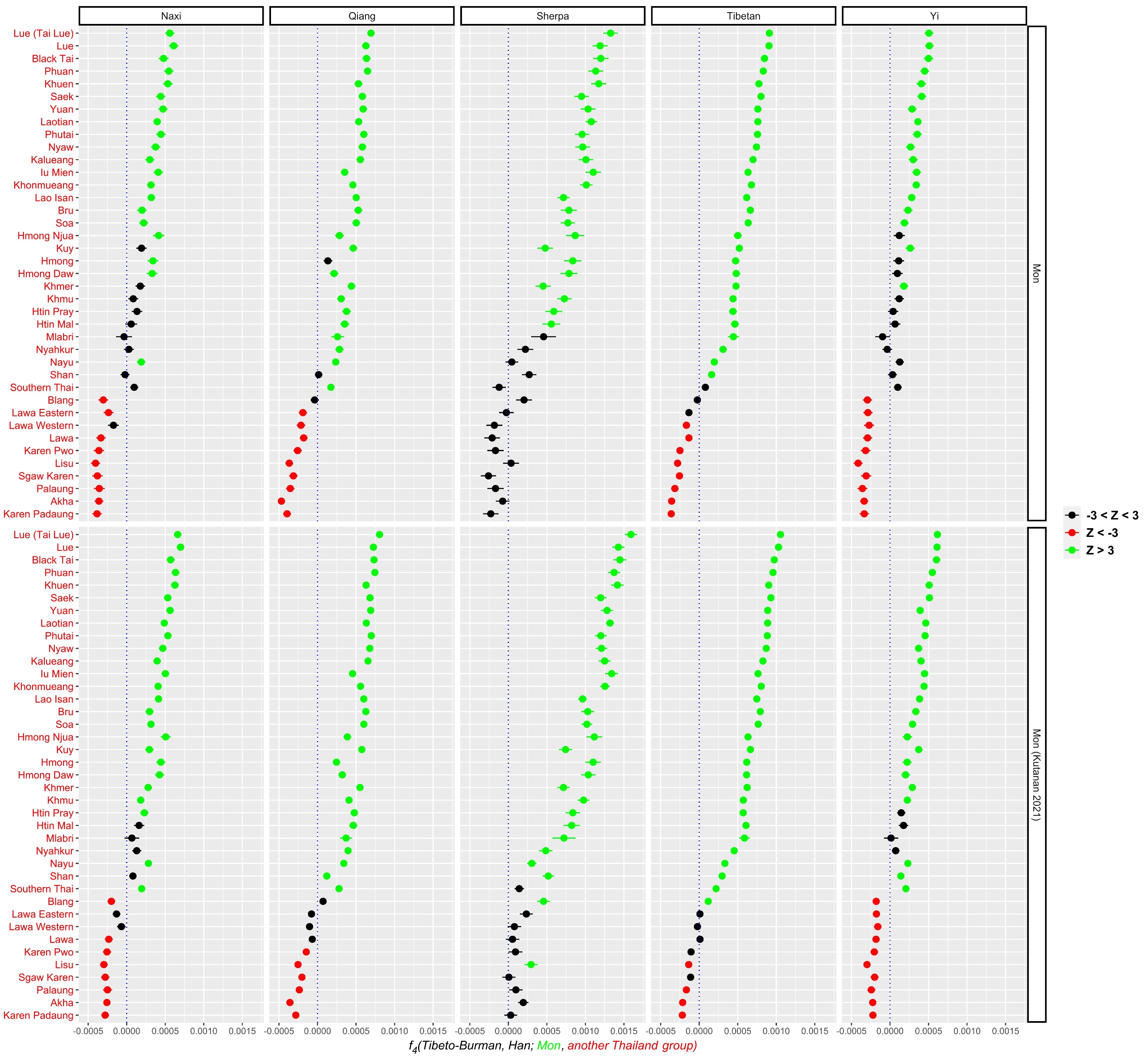


**Supplementary Figure S5.** Plot of *f*_4_ statistics value of *f*_4_(Tibeto-Burman, Han; Mons, other Thailand groups)


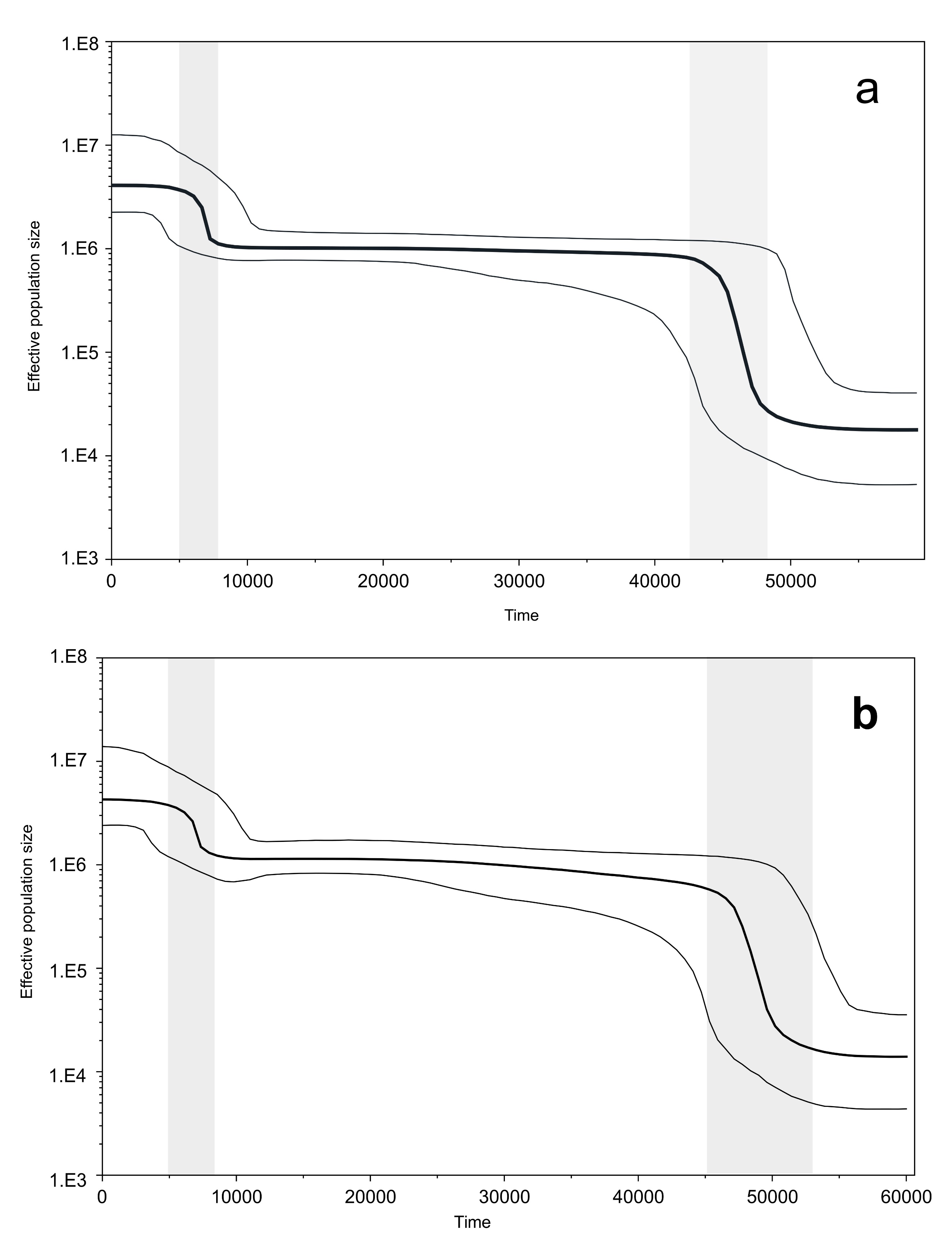


**Supplementary Figure S6.** The Bayesian skyline plot illustrates the estimated effective population size over time in years before present. The thick black line represents the median estimate, while the two thin lines correspond to the upper and lower bounds of the 95% highest posterior density (HPD) interval. The x-axis denotes the time in years before the present, and the y-axis is presented on a logarithmic scale. **(a).** the entire sequence with a mutation rate of 2.285 × 10^−8^ substitutions per site per year. **(b).** partition into coding and non-coding regions with mutation rates of 1.708 × 10^−8^ and 9.883 × 10^−8^ substitutions site^-1^year^-1^ respectively.

**Supplementary Tables:**

Supplementary Table S1. The details of 166 samples in this study include GenBank ID, haplogroup, age, sex, and geographic location.

**Supplementary Table S2.** Details of the 20 ancient samples with associated ages measured in calibrated years before present (calBP) used for tip date calibration.

**Supplementary Table S3.** The coalescent ages based on Bayesian estimation with 95% highest posterior density (HPD) for each haplogroup, estimated using two different approaches: (1) the entire mtDNA sequence with a single mutation rate of 4.33 × 10^−8^ substitutions per site per year, and (2) the mtDNA partitioned into coding and non-coding regions with mutation rates of 1.708 × 10^−8^ and 9.883 × 10^−8^, respectively. These estimates are compared to coalescent ages reported in previous studies.

**Supplementary Table S4.** Analysis of molecular variance (AMOVA) results for 166 samples grouped by 6 administrative regions.

Supplementary Table S5. The dataset for population comparison includes whole mtDNA data of 6533 samples. The dataset includes the following columns:

- accession_id (Genbank ID)
- labsample_id (internal sample name per study)
- Dataset (by publication)
- Haplogroup_published (published haplogroup)
- Haplogroup (newly assigned haplogroup based on the FASTA sequences with HaploGrep2 and Phylotree 17)
- Ethnicity (published ethnic group/group abbreviation) Population (consolidated populations based on ethnicity)
- SuperPop (super population, based on 1000 Genomes – see <https://www.internationalgenome.org/faq/which-populations-are-part-your-study/>)
- SuperPop2 (1000 Genomes super population + countries for other datasets)
- SuperPop3 (1000 Genomes super population for AMR, AFR, EUR, SAS, and populations within EAS including country-level grouping for other datasets)
- Supergroup (super Haplogroups with 1 letter + 1 number)
- FirstLetter (haplogroup first letter only except for African Lineages L0-L6)
- Phylotree (25 clades of the subtrees as listed on Phylotree website <http://phylotree.org/tree/index.htm>)
- Macrohaplogroup LMNR (macrohaplogroups grouping all L, M, N, or R clades)

**Supplementary Table S6.** Common terminal lineage haplogroups shared by Thai and South Asian populations.

**Supplementary Table S7.** Whole mtDNA evolutionary rates reported in previous studies.
